# Bacterial vaginosis, the leading cause of genital discharge among women presenting with vaginal infection in Dar es Salaam, Tanzania

**DOI:** 10.4314/ahs.v21i2.7

**Published:** 2021-06

**Authors:** Mtebe V Majigo, Paschal Kashindye, Zachariah Mtulo, Agricola Joachim

**Affiliations:** Department of Microbiology and Immunology, Muhimbili University of Health and Allied Sciences Dar es Salaam, Tanzania

**Keywords:** Bacterial vaginosis, vaginal discharge, genital infection

## Abstract

**Background:**

Pathological vaginal discharge is a common complaint of women in reproductive age worldwide caused by various agents. The prevalence and etiologic agents vary depending on the population studied. Management of vaginal discharge in low-income countries, typically depend on the syndromic approach, which limits understanding the specific causative agents. We determined the proportion of bacterial vaginosis, candidiasis, and trichomoniasis among women with vaginal discharge at a regional referral hospital in Dar es Salaam, Tanzania.

**Methods:**

We conducted a cross-sectional study between June and August of 2017 among nonpregnant women at Amana Regional Referral Hospital. Experienced staff performed physical examination to establish a clinical diagnosis, and collection of the high vaginal swab for microscopic examination. Descriptive statistics were performed to assess the characteristics of study participants and the proportion of vaginal infections.

**Results:**

A total of 196 samples were collected, of all, 128 (65.3%) had either bacterial vaginosis, candidiasis, or trichomoniasis. Bacterial vaginosis was the leading infection at 33.2%, followed by candidiasis (19.4%) and trichomoniasis (13.3%). Laboratory confirmed vaginal infection were generally found more in age below 25, unmarried, and those employed or petty business.

**Conclusion:**

The proportion of bacterial vaginosis in women with vaginal discharge was relatively higher than others, and the presence of vaginal infection relate to socio-demographic characteristics. Further advanced studies are needed to understand the potential role of aetiologic agents in causing vaginal infections.

## Introduction

Vaginal discharge affects millions of women worldwide and has been reported to be among the common complaints of women in reproductive age^1^, ^2^. About a quarter of adult women report this complaint^3^; nevertheless, the discharge may be expected due to slightly overgrowth of naturally occurring vaginal organisms^2^. Vaginal discharge categorized as pathological is characterized by the presence of greenish or yellowish, foul-smelling, and sometimes associated with itching. Bacteria, fungus, and parasites are common causes of vaginal infection associated with pathological vaginal discharge^4^, ^5^. The reported prevalence of vaginal infections varies depending on the population in which the study is conducted^5^.

The majority of vaginal infections are attributable to three causes: bacterial vaginosis (BV), candidiasis, and trichomoniasis^6^–^8^. The occurrence of BV has been associated with various factors related to intra-vaginal practices^5^, ^9^, causing depletion Lactobacilli, overgrowth of Gardnerella vaginalis, and overgrowth of anaerobic Gram-negative rods^4^, ^10^, ^11^. Studies in the sub-Saharan Africa region have reported the prevalence of BV up to 50%^9^, ^12^. Having BV and trichomoniasis in pregnancy has been linked to preterm delivery and low birth weight^13^. Also, vaginal infection is likely to increase the risk of infection with human immunodeficiency virus (HIV)^14^. Various studies have shown that women with BV are more likely to be co-infected with a sexually transmitted viral infection like herpes simplex virus type-2 and HIV^15^–^18^.

Diagnosis and treatment of BV, candidiasis, and trichomoniasis in most low-income countries rely on the syndromic approach of vaginal discharge syndrome. In Tanzanian women reporting vaginal discharge are managed using the algorithm as per national guidelines for the management of sexually transmitted and reproductive tract infections^19^. The algorithm takes into consideration both common vaginal and cervical infections. The policy requires an aetiologic/laboratory approach at facilities with well equipped functional laboratories like referral facilities. However, in practice, women who present with vaginal discharge syndrome at the outpatient and gynecologic clinic are managed following the syndromic approach. There is a possibility of overtreating a combined vaginal infection, which is mostly symptomatic and easily diagnosed microscopically.

This study determined the proportion of BV, candidiasis, and trichomoniasis, which commonly causes symptomatic vaginal infections. The findings generate data, which can be an input to review the current management approach for vaginal discharge syndrome and the prevention of complications of vaginal infections.

## Materials and methods

### Study setting

The study was conducted at amana regional referral hospital (arrh) in dar es salaam Tanzania. ARRH serves the population of Ilala Municipal with an approximate of 1,220,611 people as of 2012 census. The hospital has an authorized bed capacity of 362; however, the outpatient attendance ranges between 800–1200 daily. The hospital receives referred cases from the health centers around Ilala Municipal, sometimes from dispensaries and client self-referral.

### Study design

This was a cross-sectional study conducted between June and August 2017 at ARRH in Dar es Salaam, Tanzania. Enrolled participants were examined to establish the clinical diagnosis of vaginal discharge syndrome and investigated for the presence of BV, candidiasis, and trichomoniasis.

### Study population

The study involved women presenting with signs and symptoms suggestive of vaginal infections at outpatient and gynecological clinics. The symptoms included perceived abnormal vaginal discharge which has different color, odor, consistency or amount than normal discharge and vaginal itching.Routinely, similar cases are examined by attending clinicians to establish the clinical diagnosis of vaginal discharge syndrome before initiation of treatment. For this study, women with symptoms of vaginal discharge syndrome and observed abnormal vaginal discharge and vaginal wall inflamantion the features suggestive of vaginal infection upon physical examination were requested to participate in the study. The study enrolled participants after provide written consent for the collection of high vaginal swab specimens. The study included women with age of 18 years and above excluding women who were pregnant, menstruating, and who had started antibiotics

### Sample size and sampling technique

The sample size was estimated using Kish Leslie formula^20^, at a 95 % confidence interval considering 15% prevalence of pathological vaginal discharge among women at the gynecology clinic^7^ and a 5 % margin of error. Eligible clients were consecutively enrolled in the study until reaching a representative sample size.

### Specimen and Data collection

A high vaginal swab specimen was aseptically collected by attending clinician or specialized nurse. A standard speculum examination was performed for each participant using sterile Cusco's speculum without lubricant. Macroscopic evaluation of the vaginal walls for color, amount and consistency of the discharge were noted A sterile cotton swab (H-Media Ltd, India) was used to collect specimens from the posterior fornix of the vagina and then placed in commercially available Amies transport medium (H-Media Ltd, India), labeled with the specific identification number. Specimens were transported at ambient temperature to the Microbiology laboratory at the Muhimbili University of Health and Allied Sciences for further processing within 12 hours. Participants' information regarding socio-demographic characteristics, including age, marital status, occupation, and level of education were obtained from patients' files and laboratory request form.

### Laboratory investigations

Wet-mounts made from the swabs in transport media were examined for motile trichomonads by light microscopy using high dry power (x 40). The diagnosis of trichomoniasis was reached after detecting Trichomonas vaginalis, recognized by their jerky movements, undulating membrane, and possession of four flagella.

The smears were made, Gram-stained, and scored for BV by an experienced laboratory technologist according to Nugent's criteria as previously described^21^. Three types of bacterial morphotypes were evaluated: large gram-positive rods, small gram-negative/variable rods, and curved gram variable rods. A Nugent score of 0–3 was interpreted as negative, a score of 4–6 as intermediate, while a score of 7–10 was interpreted as consistent with BV.

The same Gram-stained smears were examined for the presence of Gram-positive budding yeast cells. A diagnosis of *Candida* spp as the etiological agent was based on the presence of Gram-positive yeast cells and a clinical diagnosis of vulvovaginal candidiasis and thick curdy vaginal discharge.

### Quality control

The laboratory procedures were performed by following the established standard operating procedures. Quality assurance procedures were set during specimen collection, smear preparation, staining, and microscopic examination. Quality of stains was tested on each day of use with both positive and negative control smears for BV and *Candida* spp.

### Data analysis

Conventional descriptive statistics were used to assess the characteristics of study participants and the proportion of vaginal infections. A Chi-square test was used to determine whether there were significant differences between observed proportions of vaginal infections in the studied population. The differences of socio-demographic characteristics with BV, candidiasis, and trichomoniasis were made using the Pearson Chi-square test with a test of significance being two-tailed (p < 0.05). Data were analyzed using Statistical Package for Social Sciences version 20.0

## Results

### Socio-demographic characteristics

A total of 196 participants had high vaginal swabs specimen collected; the median age was 29 years, 25.0 – 34.7 years inter-quartile range (IQR). The majority, 111(56.6%) were aged between 25–35 years, and 137(69.9%) had low education (primary obelow education level). More than half of participants, 116 (59.2%) engaged in petty businesses (independently owned small business) while 151 (77.0%) were married (Table 1)

**Table 1:** Socio-demographic characteristics of 196 study participants

Characteristics	Frequency	Percentages (%)
**Age group**		
< 25	43	21.9
25 – 35	111	56.6
> 35	42	21.4
**Education**		
Low	137	69.9
High	59	30.1
**Occupation**		
Housewife	68	34.7
Petty business	116	59.2
Employed	12	6.1
**Marital status**		
Married	151	77.0
Single	45	23.0

### Vaginal infections

Of all, 128 (65.3%, 95% CI, 58.4–71.6) had one of the three vaginal infections (BV, candidiasis, trichomoniasis)). BV was the leading infection with 65(33.2%, 95% CI, 26.9–40.0) followed by candidiasis (38(19.4%, 95% CI, 14.4–25.5). Mixed infection was observed only in 1(0.5%) participant having candidiasis and trichomoniasis (Table 2)

**Table 2:** Proportion of participants with positive results of vaginal infection among 196 Participants

Infection	Proportion (%)	95% CI of Proportion
Total vaginal infection	128 (65.3)	58.4–71.6
Bacterial vaginosis	65 (33.2)	26.9–40.0
Candidiasis	38 (19.4)	14.4–25.5
Trichomoniasis	26 (13.3)	09.2–18.7
Candidiasis + Trichomoniasis	1(0.5)	

### Vaginal infection a nd socio-demographic Characteristics

The differences in vaginal infection with socio-demographic characteristics are shown in table 3. Patients dealing with the petty business had a significantly higher proportion of BV (46.6%) than other occupations (p < 0.01). Those who were married had more BV (38.4%) compared to single 7(15.6%) (p < 0.01). There was no difference in the proportion of BV in different education levels and age groups.

**Table 3:** Bacterial vaginosis, Candidiasis, and trichomoniasis and sociodemographic characteristics of study participants

variable	B. Vaginosis		Candidiasis		Trichomoniasis		Vaginal infection*	
	
	N (%)	*p value*	N (%)	*p value*	N (%)	*p value*	N (%)	*p value*
**Age (years)**		0.89		0.44		**< 0.01**		**0.042**
Below 25	14(32.6)		8(18.6)		13(30.2)		35(81.4)	
25 – 35	36(32.4)		19(17.1		13(11.7)		67(60.4)	
Above 35	15(35.7)		11(26.2)		0		26(61.9)	
**Marital Status**		**0.004**		0.46		**< 0.001**		**0.007**
single	7(15.6)		7(15.6)		24(53.3)		37(82.2)	
Married	58(38.4)		31(20.5)		2(1.3)		91(60.3)	
**Occupation**		**< 0.001**		**< 0.001**		**0.002**		**<0.001**
Housewife	8(11.8)		19(27.9)		1(1.5)		28(41.2)	
Petty business	54(46.6)		13(11.2)		23(19.8)		89(76.7)	
Employed	3(25.0)		6(50.0)		2(16.7)		11(91.7)	
**Education**		0.71		0.57		0.70		0.878
Low level	43(31.4)		28(20.4)		19(13.9)		89(65.0)	
High level	22(37.3)		10(16.9)		9(11.9)		39(66.1)	

The significant difference of candidiasis in different socio-demographic characteristics was observed only for types of participant's occupation. Employed participants had more infection with candidiasis (50%) than Housewife (27.9%), and Petty business (11.2%), p < 0.01).

Trichomoniasis was observed more in the age group below 25 years (30%), single marital status (53.3%), and petty business (19.8%) than other characteristics in the same categories, and the differences were statically significant (p < 0.01). There was no sinificant difference proportion of any vaginal infections with the level of educations. There was an association between the presence of at least one vaginal infection and age group, marital status as well as an occupation (p < 0.05) (Table 3)

## Discussion

The current study on vaginal infection among women with vaginal discharge reports a relatively higher proportion of BV compared to candidiasis and trichomoniasis. Almost every individual except one had a single infectious condition of the three common causes of vaginal infection. About one-third of participants had unidentified etiology of vaginal discharge. These findings necessitate the need for looking into other etiological agentsthat may lead to vaginitis since etiologies of genital tract infections may vary widely.^22^. The finding of BV being the most common etiologic condition of vaginal discharge followed by candidiasis was coherent with findings from other studies investigating vaginal infection^23^–^25^. Our findings, however, differ from the finding of previous research in the same location^9^. Candidiasis, followed by BV as the most prevalent vaginal infection, was reported in studies conducted in Ethiopia and India^8^, ^26^. The differences observed in different studies can be explained by the difference in the actual study participants, personal hygiene practice, environment, socioeconomic and cultural factors of the study participants.

The prevalence of BV in the present study was comparable with various studies that reported a relatively high prevalence of BV, ranging from 26% to 34%^27^–^29^. However, some studies have reported a low prevalence of BV to compare to our findings^7^, ^30^, ^31^. The variations in the prevalence of BV reported in different studies may be attributed by differences in study population and method used for the detection of bacterial vaginosis^26^,^32^, ^33^. The magnitude of BV in this study might indicate a high rate of disturbance of normal genital microbial flora that led to the proliferation of pathogenic organisms. Women in this community might be practicing intravaginal practices as the previous report indicated a high rate of vaginal douching associated with increased prevalence of BV9.

Trichomoniasis was the least observed in our study compared to other vaginal infections but slightly higher than earlier reports in the same geographical location9 and other parts of the world^26^, ^34^, ^35^. A study done among pregnant women in Botswana reported a higher prevalence of 19% for trichomoniasis^36^. The differences of trichomoniasis found in different studies could be due to variations in personal hygiene practice, socioeconomic, and cultural factors of the study participants. Moreover, the detection of Trichomonas vaginalis by the conventional wet-mount method in the present study might have reduced the actual prevalence^37^.

The proportion of vaginal infection by either BV, Candidiasis, or trichomoniasis was found to vary in participants' marital status, occupation, and age groups. Married participants had a significantly higher proportion of BV. The finding is coherent with a study done in Egypt^27^. On the contrary, the study done in Nepal found that unmarried women were at higher risk compared to married women^38^. Likewise, BV has been found in sexually inactive females^39^,^40^, signifying the presence of other factors rather than sexual activity alone as the risk for acquisition of BV. In the present study, trichomoniasis was observed more in the age group below 25 years and those who were single. Our finding of age in relation to trichomoniasis was comparable to a study done in Ethiopia^7^ and Iran^35^.

This was a small study with limited data for factors contributing to vaginal infection and for evidently establish the reason for different observations found among participants, even though some findings are coherent or contrary to other studies. In this study, we relied on microscopic methods of detecting BV, candidiasis, and trichomoniasis. Using the culture method could have been more accurate than the microscopic examination.

## Conclusion

There is a relatively high proportion of bacterial vaginosis among nonpregnant women and could be affected by certain socio-demographic characteristics. We recommend further longitudinal and follow-up studies to investigate the effect of infection and the role of BV, candidiasis, and trichomoniasis in causing vaginal infections.

## Abbreviations

ARRH: Amana Regional Referral Hospital, BV: bacterial vaginosis, HIV: human immunodeficiency, VC: vaginal candidiasis, TV: *Trichomonas vaginalis*
